# Editorial: Nutritional factors and interventions in environmental-borne diseases

**DOI:** 10.3389/fnut.2023.1241030

**Published:** 2023-07-12

**Authors:** Reinaldo B. Oriá, Ronald F. Pinheiro, Jacqueline I. Alvarez-Leite

**Affiliations:** ^1^Laboratory of Tissue Healing, Ontogeny, and Nutrition, Department of Morphology, School of Medicine, Institute of Biomedicine, Federal University of Ceara, Fortaleza, Brazil; ^2^Cancer Cytogenomics Laboratory, Drug Research and Development Center, Federal University of Ceara, Fortaleza, Brazil; ^3^Laboratory of Atherosclerosis and Nutritional Biochemistry, Department of Biochemistry and Immunology, Federal University of Minas Gerais, Belo Horizonte, Brazil

**Keywords:** environmental enteric dysfunction (EED), pollutants, congenital heart defect, metabolic syndrome risk factor, malnutrition, trace elements, oxidative stress, insulin resistance

Environmental-borne diseases are a public health concern affecting vulnerable populations due to the increased disarray of urbanization, industrialization, and climate change, which are unprecedentedly challenging human living conditions worldwide. Often seen in the developing world, poverty and inadequate hygiene and sanitation increase endemic enteric pathogens exposure, causing environmental enteropathy/environmental enteric dysfunction (EED), with disturbances of the gut–brain axis and distinct degrees of intestinal and brain inflammation. Even at low levels, soil, air, water, and food contaminants/pollutants that can bioaccumulate in the environment are known to affect normal human development and aging deleteriously.

In our Research Topic, we gathered four outstanding papers addressing the role of environmental-related factors and disease conditions with innovative contributions to the field of nutritional sciences.

Kabir et al. studied the impact of co-colonization of EED-related enteric pathogens in non-diarrheal stools and their association with fecal and intestinal inflammatory biomarkers on growth delta *z*-scores and velocity from Pakistani children during their first 2 years of life as part of the “Study of Environmental Enteropathy and Malnutriton (SEEM).” They also evaluated the distribution of antimicrobial resistance (AMR) genes (*ctxM, gyrA*, and *parC*).

The authors found a high prevalence of *Giardia, Campylobacter* spp., enterotoxigenic, enteropathogenic, and enteroaggregative *Escherichia coli, Cryptosporidium* spp., and norovirus in the non-diarrheal stool samples. Recurrence of fecal contamination was seen with *Giardia, Campylobacter*, enterotoxigenic *E. coli, Shigella*, and *Cryptosporidium*. *Giardia* presence in non-diarrheal stools was nearly ubiquitous and positively correlated with fecal lipocalin, a bacteriostatic compound, at 3 and 6 months and alpha-1-acid glycoprotein (AGP) at 9 months. *Campylobacter* spp. positively correlated with neopterin (NEO) and myeloperoxidase (MPO). Protozoa occurrence in stools at 6 months was negatively correlated with delta height-per-age *z*-scores at 12 and 24 months. Notably, co-colonization of Giardia and Campylobacter was confirmed from the non-diarrheal stools collected at 9 months, with a significant impact noticed on delta growth measures. Regarding antimicrobial-resistant genes, at least one studied gene was found in more than 75% of the samples, mainly *ctxM*, related to β-lactam antibiotics use. This finding may partly explain why antibiotic trials have failed to improve the deleterious effects of enteric pathogens on children's growth.

Liang et al. elegantly revised data from case-control, animal, and *in vitro* studies about the impact of exposure to essential and non-essential trace elements on congenital heart defect (CHD) risk, highlighting epidemiologic, preventive, and pathogenetic aspects. Although CHDs have been partly remedied by early diagnosis and surgery, they remain an important cause of health burden and mortality, especially in the developing world. In fact, not only deficiency but also the excess of trace and non-trace elements may cause CHDs. The mechanisms associated with abnormal heart development include oxidative stress and mitochondrial dysfunction. CHDs are associated with the altered expression of genes regulating cardiomyocyte viability and differentiation. Moreover, the authors discussed the importance of understanding trace and non-trace elements' accumulative, synergistic, and additive effects as important players during heart development and as contributors to CHDs. The supplementation of these elements during pregnancy, especially for Cu, Zn, and Fe, has a beneficial role during heart development and calls attention to avoiding heavy metal exposure.

Zamora et al. evaluated the metabolic syndrome risk score (MRS) and oxidative stress biomarkers in 250 adolescents prenatally exposed to bisphenol A (BPA) from mothers from low- and moderate-income backgrounds who adhered to a routine Mediterranean diet. Data were obtained from two prospective birth cohorts from Early Life Exposure in Mexico to Environmental Toxicants (ELEMENT). The results revealed that maternal weight gain was positively associated with MRS in female adolescents. Moreover, MRS was positively associated with age and stage of puberty in both sexes and leptin levels.

However, none of the maternal or adolescent characteristics alone affected levels of 8-isoprostane (8-iso), an oxidative stress biomarker. When data were stratified by sex, an increase in prenatal BPA exposure during the second trimester was surprisingly associated with a decrease in MRS for male adolescents. This association was not yet totally understood.

The authors showed that an increased BPA exposure during the second trimester led to an increase in 8-iso levels. However, this was only significant in female adolescents, with significant improvement in adherents to the Mediterranean diet (MD). This suggests that BPA exposure associated with MD in the second trimester led to sexually dimorphic effects on the adolescent offspring's oxidative stress and metabolic syndrome risk. This was the first human epidemiologic study examining the impact of prenatal programming on metabolic syndrome outcomes of offspring exposed to endocrine-disrupting chemicals.

Gayathri et al. conducted a randomized, controlled trial to assess the efficacy of almonds, rich in unsaturated fatty acids and micronutrients, in reducing insulin resistance and improving the lipid profile of overweight Asian Indian adults. Unhealthy diets and sedentarism are critical contributors to the risk of obesity, diabetes, and cardiovascular diseases. The participants consumed 43 g of almonds/day for 12 weeks. Almond-supplemented individuals reduced body weight, waist circumference, and body mass index, as well as insulin resistance and cholesterolemia, compared to the controls (avoiding nuts). The almond-supplemented group also presented higher fiber and healthy fats (poly- and monounsaturated fatty acids) intakes, with a decrease in carbohydrate intake. Although this study was restricted to Asian Indians, it had a large cohort of 400 participants, providing evidence that healthy diet changes can reduce the risk of developing chronic diseases.

In summary, this Research Topic provides an excellent contribution to understanding the effects of environmental biohazards on the risk of disease and its progression, calling for awareness of the need to successfully build nutritional interventions for prevention and treatment strategies.

## Author contributions

All authors listed have made a substantial, direct, and intellectual contribution to the work and approved it for publication.

